# Low muscle density in children with osteogenesis imperfecta using opportunistic low-dose chest CT: a case-control study

**DOI:** 10.1186/s12891-024-07596-7

**Published:** 2024-06-18

**Authors:** Yi Yuan, Yun-feng Xu, Chao Feng, Yan-dong Liu, Wen-shuang Zhang, Peng-ju Huang, Kang-kang Ma, Feng-yun Zhou, Zi-tong Cheng, Zheng Yang, Ling Wang, Xiao-guang Cheng

**Affiliations:** 1 Department of Radiology, Beijing Jishuitan Hospital, Capital Medical University, National Center for Orthopaedics, No. 31, Xinjiekou East Street, Beijing, 100035 China; 2 Department of Pediatric Orthopaedics, Beijing Jishuitan Hospital, Capital Medical University, National Center for Orthopaedics, No. 31, Xinjiekou East Street, Beijing, 100035 China

**Keywords:** Osteogenesis imperfecta, Child, Computed tomography, Muscles

## Abstract

**Background:**

The aim of the study was to investigate the muscle differences in children with osteogenesis imperfecta (OI) using opportunistic low-dose chest CT and to compare different methods for the segmentation of muscle in children.

**Methods:**

This single center retrospective study enrolled children with OI and controls undergoing opportunistic low-dose chest CT obtained during the COVID pandemic. From the CT images, muscle size (cross-sectional area) and density (mean Hounsfield Units [HU]) of the trunk muscles were measured at the mid-T4 and the mid-T10 level using two methods, the fixed thresholds and the Gaussian mixture model. The Bland-Altman method was also used to compute the strength of agreement between two methods. Comparison of muscle results between OI and controls were analyzed with Student *t* tests.

**Results:**

20 children with OI (mean age, 9.1 ± 3.3 years, 15 males) and 40 age- and sex-matched controls were enrolled. Mean differences between two methods were good. Children with OI had lower T4 and T10 muscle density than controls measured by the fixed thresholds (41.2 HU vs. 48.0 HU, *p* < 0.01; 37.3 HU vs. 45.9 HU, *p* < 0.01). However, children with OI had lower T4 muscle size, T4 muscle density, T10 muscle size and T10 muscle density than controls measured by the Gaussian mixture model (110.9 vs. 127.2 cm^2^, *p* = 0.03; 44.6 HU vs. 51.3 HU, *p* < 0.01; 72.6 vs. 88.0 cm^2^, *p* = 0.01; 41.6 HU vs. 50.3 HU, *p* < 0.01, respectively).

**Conclusions:**

Children with OI had lower trunk muscle density indicating that OI might also impair muscle quality. Moreover, the fixed thresholds may not be suitable for segmentation of muscle in children.

## Background

Osteogenesis imperfecta (OI) is a clinically heterogeneous heritable disorder with increased bone fragility, low bone mass and increased risk of fractures [1]. OI is usually caused by autosomal dominant mutations in the type I collagen genes, COL1A1 and COL1A2, resulting in qualitative or quantitative defects of type I collagen [2]. Muscle and bone are closely linked through biomechanical forces and biochemical paracrine and endocrine factors [3]. Meanwhile, bone disorders can affect the muscles in different ways, for example, TGFbeta signaling appears to be increased in OI, and release of TGFbeta from bone can decrease muscle mass [4]. While the most conspicuous feature of OI is bone fragility and skeletal dysplasia, several studies have demonstrated that muscle weakness remains an important concern in mild-to-moderate OI patients relative to healthy individuals [5–8]. Children with OI had a low muscle size at the forearm and a muscle function deficit in the lower limb [6–8]. In addition, the exercise tolerance and muscle strength were significantly reduced in children with OI [9]. However, further exploration found that 80% of OI patients experience inherent muscle weakness instead of reduced activity [10]. Meanwhile, adults with OI had lower lean mass, muscle size, and muscle density compared to healthy controls and presented with markedly impaired muscle function [11]. Therefore, besides bone fragility, OI may be correlated with low muscle mass and function.

CT can be used to measure muscle size (cross-sectional area) and muscle density (mean Hounsfield Units [HU]) of the abdominal and mid-thigh muscles [12, 13]. Muscle density measured by CT as the mean attenuation in HU and has been regarded as a marker of muscle quality [14]. Low-dose chest CT scans can be performed for screening pneumonia in children with OI with less ionizing radiation and CT is the best way to reconstruct the complex morphology of the internal skeletal thoracic anatomy [15]. However, due to the relative high radiation dose of chest CT, acquisitions of CT-based muscle assessments were almost not available in children with or without OI. Thus, little is known about the muscle quality of children with OI. Furthermore, the CT value of muscle will vary considerably depending on its adipose tissue content within a single muscle or within a muscle group contained by the perimuscular deep fascia and the use of absolute thresholds is problematic [12]. They also do not take into account deviations from the ideal water calibration of the CT scanner [12]. A consensus of an attenuation range has not been reached for skeletal muscle attenuation and differential range utilization in image analysis may have a conspicuous impact on the calculation of muscle size [16]. For example, between edema and washed out muscle structures, CT value differences are small and therefore techniques based on the fixed thresholds to initiate the segmentation will have severe limitations [17]. To our knowledge, little is known about the proper thresholds for segmentation of muscle in children.

The primary purpose of the present study was to investigate trunk muscle size and density in children with OI by using opportunistic low-dose chest CT screening and to compare muscle measurements in OI children with those in age- and sex-matched controls. We hypothesized that children with OI may have a smaller muscle size and lower muscle density in the trunk muscles compared with age- and sex-matched controls. In addition, the purpose of the present study was also to compare and evaluate two methods and determine whether the fixed thresholds was suitable for the segmentation of muscle in children.

## Materials and methods

### Participants

This retrospective study recruited children with OI and controls admitted to department of pediatric orthopaedics between April 2020 and July 2022 in our hospital. The children with OI and controls were hospitalized because of fractures in different sites that required the surgery. During the COVID pandemic period, opportunistic low-dose chest CT scans should be performed for screening pneumonia before surgery. Inclusion criteria were applied: (1) a clinical diagnosis of OI; (2) availability of opportunistic low-dose chest CT results. Exclusion criteria included: (a) insufficient image quality due to severe movement artifacts; (b) loss of opportunistic low-dose chest CT images. Controls were matched to patients by age and sex. Exclusion criteria of the controls was history of receiving bisphosphonate treatment.

### Ethical consideration

This study was approved by the Ethics Committee of Beijing Jishuitan Hospital (approval No. K202219900). It was performed in line with the principles of the Declaration of Helsinki. Written informed consent was obtained from parents/legal guardian of all the participants included in the study.

### Low-dose chest CT acquisition

Low-dose chest CT was performed for all study participants using a TOSHIBA Aquilion PRIME TSX-302 A scanner (Toshiba Medical System Division, Tokyo, Japan, 2015). Scans were acquired from the apical lung to the lower edge of L2. According to different weights and ages, scan parameters were the range of 80-120kVp, 30-100mAs, 40 cm field of view, 1 mm standard reconstruction interval, and a standard reconstruction kernel with adaptive iterative dose reduction.

### Muscle parameters measurements

**Fig. 1:**
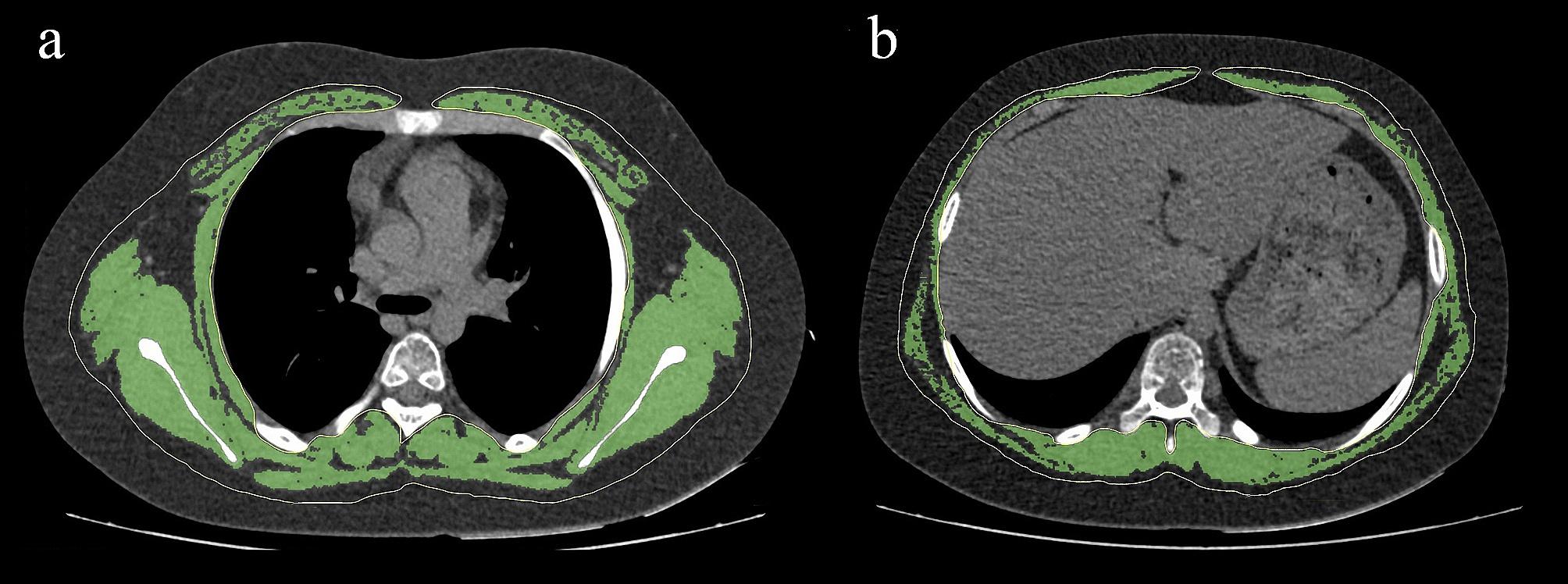
Measurement of cross‒sectional area and mean CT values of the trunk muscles. Measurement of cross‒sectional area and mean CT values of the trunk muscles at the level of the mid-T4 (**a**). Measurement of cross‒sectional area and mean CT values of the trunk muscles at the level of the mid-T10 (**b**)

Cross-sectional area and density of the following trunk muscles or muscle groups were measured on 1 slice each at the mid-T4 and the mid-T10 level. Mid-T4 was defined as the slice including the middle of 4th thoracic vertebrae. Mid-T10 was defined as the slice including the middle of 10th thoracic vertebrae. At the mid-T4 level, thoracic and back (pectoralis, intercostalis, paraspinals, serratus, latissimus dorsi) muscles were measured. At the mid-T10 level, thoracic and back (latissimus dorsi, intercostalis, serratus, paraspinals, trapezius) muscles were measured (Fig. 1).

OsiriX software (Vision 10.0.2; Pixmeo, Geneva, Switzerland) was used for analysis. Multi-planar reconstruction of the vertebral axial plane was obtained by using OsiriX. Muscle segmentation was drawn manually using the “pencil” tool to outline muscle contours. Then the 2-dimensional/3-dimensional segmentation module was used to semiautomatically select skeletal muscle regions within the fixed HU intensity thresholds used in adults (-30 to 150 HU). A threshold of -29 HU was used to segment muscle tissue from fat [18]. Muscle cross-sectional area (cm^2^) was calculated by subtracting the bone cross-sectional area from the combined muscle and bone cross-sectional area. Muscle density (HU) was calculated as the mean density of the tissue in the muscle cross-sectional area [7].

**Fig. 2:**
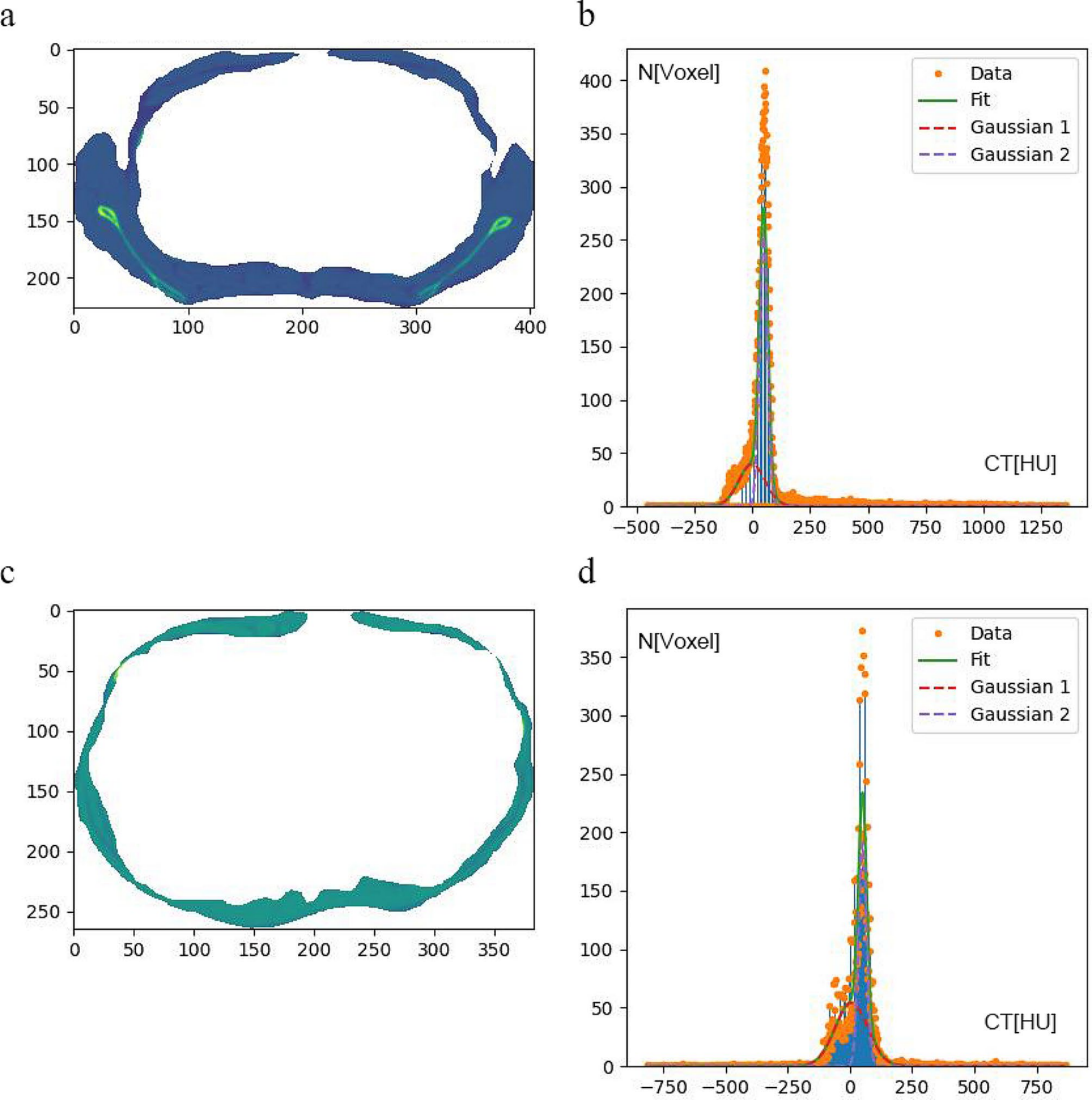
Image-specific definition of thresholds for anatomical muscle and muscle tissue. The trunk muscles at the level of the mid-T4 and the mid-T10 (**a, c**). The Gaussian mixture model used to fit adipose (red) and muscle tissue (purple) distributions (**b, d**)

For the definition of muscle, the Gaussian mixture model was employed in combination with a Levenberg-Marquard optimization algorithm [17] to fit two Gaussian curves to the CT value spectrum of the combined adipose tissue and intrafascia. One fit curve represented adipose tissue and muscle tissue. For muscle tissue the ratio of the number of voxels of adipose tissue versus those of muscle-lipid system was used to initialize the height. Muscle tissue was then defined by 3D volume growing inside intrafascia starting from seed points defined as voxels with CT values higher than the peak b of the fitted muscle tissue curve (Fig. 2).

### Data collection

Demographic variables including gender, age, weight, height, and body mass index (BMI) and other health-related data were retrieved from the patient’s medical file or from the participants’ medical records.

### Statistical analysis

Statistical analyses were calculated by using SPSS 23.0 (IBM, Armonk, NY, USA). The Bland-Altman method was also used to compute the strength of agreement between two methods for muscle measurements. The range of limit of agreement indicates an interval that comprises 95% of the differences between two methods. Differences between OI patients and controls were analyzed using a Student *t* test for continuous variables and the χ^2^ test for categorical variables. Differences between clinical data and muscle parameters were analyzed using a Student *t* test and ANOVA analysis for continuous variables in OI patients. Differences between clinical data and muscle parameters were analyzed using a Student *t* test for continuous variables in controls. *p* < 0.05 was considered significant.

## Results

### Mean differences between two methods

**Fig. 3:**
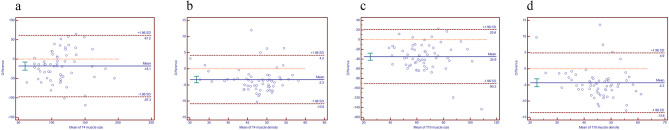
Bland-Altman plots of mean differences for two methods for muscle measurements. Bland-Altman plots of mean differences of T4 muscle size (**a**), T4 muscle density (**b**), T10 muscle size (**c**) and T10 muscle density (**d**). Solid horizontal blue line indicates mean difference. The upper and lower dashed lines correspond to upper and lower 95% limits of agreement which are calculated by mean differences ± 1.96 standard deviation

Table 1 shows mean differences of muscle measurements using two methods. The Bland-Altman plots represents the relationship between the differences and mean muscle measurements measured by the fixed thresholds and the Gaussian mixture model illustrated in Fig. 3. Two methods are considered to have good agreement in muscle density when the difference is small enough for both methods to be used interchangeably. Because the error is normally distributed, the majority of points were between the 95% limits of agreement range.

**Table 1:** Mean differences of muscle measurements between two methods

Muscle parameters	Mean difference	95% limits of agreement
T4 muscle size (cm^2^)	-18.05	-97.30-61.19
T4 muscle density (HU)	-3.34	-10.84-4.16
T10 muscle size (cm^2^)	-34.94	-90.51-20.62
T10 muscle density (HU)	-4.34	-13.59-4.90

Note: T4, thoracic vertebra; T10, thoracic vertebra

### Comparison between patients with OI and control

**Fig. 4:**
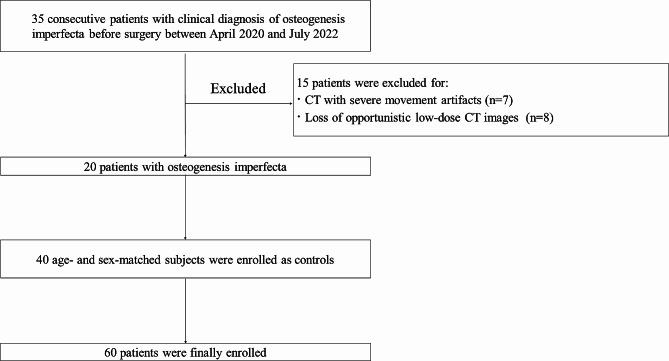
Flow diagram shows inclusion and exclusion criteria for the study

Results are summarized in Table 2 for clinical characteristics and muscle parameters between patients with OI and control group. Of 35 consecutive children with OI admitted to the hospital, 20 patients consisted of 15 males and 5 females were enrolled. Seven patients had poor image quality and 8 patients lost CT images were excluded (Fig. 4). The children with OI ranged in age from 3 years to 13 years, with a median of 10 years. Of the 40 controls, 30 were males and 10 were females. The children ranged in age from 3 years to 14 years, with a median of 10 years. The height of controls was significantly higher than that of OI individuals (144.6 vs. 131.7 cm, *p* = 0.04).

**Table 2:** Differences between OI patients and controls

	OI (*n* = 20)	Controls (*n* = 40)	*p* Value
Gender			0.63
Male	15(0.750)	30(0.750)	
Female	5(0.250)	10(0.250)	
Age	9.1 ± 3.3	9.7 ± 3.0	0.47
Weight (kg)	38.4 ± 24.1	43.7 ± 18.8	0.35
Height (cm)	131.7 ± 26.8	144.6 ± 19.0	0.04
BMI (kg/m^2^)	19.8 ± 4.6	20.0 ± 4.9	0.91
Muscle parameters measured by the fixed thresholds			
T4 muscle size (cm^2^)	93.1 ± 33.4	108.9 ± 34.2	0.09
T4 muscle density (HU)	41.2 ± 5.5	48.0 ± 4.7	< 0.01
T10 muscle size (cm^2^)	41.7 ± 15.5	51.0 ± 18.9	0.06
T10 muscle density (HU)	37.3 ± 6.8	45.9 ± 6.2	< 0.01
Muscle parameters measured by the Gaussian mixture model			
T4 muscle size (cm^2^)	110.9 ± 18.4	127.2 ± 36.8	0.03
T4 muscle density (HU)	44.6 ± 7.3	51.3 ± 4.3	< 0.01
T10 muscle size (cm^2^)	72.6 ± 16.4	88.0 ± 23.8	0.01
T10 muscle density (HU)	41.6 ± 10.4	50.3 ± 6.3	< 0.01

Note: OI, osteogenesis imperfecta; BMI, body mass index; T4, thoracic vertebra; T10, thoracic vertebra

Compared with the control group, children with OI had lower T4 muscle density and lower T10 muscle density measured by the fixed thresholds (41.2 HU vs. 48.0 HU, *p* < 0.01; 37.3 HU vs. 45.9 HU, *p* < 0.01). There were no significant differences in T4 muscle size and T10 muscle size between OI patients and control group (93.1 vs. 108.9 cm^2^, *p* = 0.09; 41.7 vs. 51.0 cm^2^, *p* = 0.06, respectively). However, children with OI had lower T4 muscle size, lower T4 muscle density, lower T10 muscle size and lower T10 muscle density than controls measured by the Gaussian mixture model (110.9 vs. 127.2 cm^2^, *p* = 0.03; 44.6 HU vs. 51.3 HU, *p* < 0.01; 72.6 vs. 88.0 cm^2^, *p* = 0.01; 41.6 HU vs. 50.3 HU, *p* < 0.01, respectively).

### Differences of muscle parameters in OI individuals and in controls

Table 3 shows the gender- and OI type-specific differences of muscle parameters in OI patients and in controls by using the fixed thresholds. There were no significant differences in muscle size and muscle density between males and females. There were no significant differences in muscle size and muscle density among OI types.

**Table 3:** Differences of muscle parameters in OI patients and in controls using the fixed thresholds

	T4 muscle size (cm^2^)	T4 muscle density (HU)	T10 muscle size (cm^2^)	T10 muscle density (HU)
OI				
Gender				
Male (*n* = 15)	98.5 ± 31.8	41.2 ± 6.3	44.8 ± 15.3	36.4 ± 6.9
Female (*n* = 5)	77.1 ± 36.6	41.2 ± 2.7	32.3 ± 13.3	40.1 ± 6.3
*p* Value	0.22	0.99	0.12	0.30
OI type				
I (*n* = 15)	97.9 ± 36.2	40.4 ± 5.8	44.4 ± 16.7	36.4 ± 6.5
III (*n* = 2)	98.1 ± 18.5	43.6 ± 1.6	39.9 ± 7.2	35.5 ± 9.5
IV (*n* = 3)	66.2 ± 2.6	43.4 ± 6.1	29.3 ± 5.3	43.3 ± 5.3
*p* Value	0.33	0.59	0.32	0.27
Controls				
Gender				
Male (*n* = 30)	113.2 ± 36.0	48.1 ± 5.0	53.1 ± 20.3	46.3 ± 6.9
Female (*n* = 10)	96.2 ± 25.4	47.7 ± 3.9	44.7 ± 12.1	44.8 ± 3.7
*p* Value	0.18	0.84	0.13	0.39

Note: OI, osteogenesis imperfecta; T4, thoracic vertebra; T10, thoracic vertebra

Table 4 shows the gender- and OI type-specific differences of muscle parameters in OI patients and in controls using the Gaussian mixture model. No significant differences were found in muscle size and muscle density between males and females. No significant differences were found in muscle size and muscle density among OI types.

**Table 4:** Differences of muscle parameters in OI patients and in controls using the Gaussian mixture model

	T4 muscle size (cm^2^)	T4 muscle density (HU)	T10 muscle size (cm^2^)	T10 muscle density (HU)
OI				
Gender				
Male (*n* = 15)	110.9 ± 17.1	44.0 ± 8.2	71.0 ± 17.3	40.3 ± 10.7
Female (*n* = 5)	110.7 ± 24.0	46.2 ± 4.2	77.2 ± 13.5	45.6 ± 9.6
*p* Value	0.98	0.45	0.48	0.34
OI type				
I (*n* = 15)	111.4 ± 15.1	43.4 ± 7.8	73.0 ± 15.2	39.9 ± 10.4
III (*n* = 2)	123.7 ± 26.6	47.4 ± 0.4	64.7 ± 19.3	40.6 ± 8.5
IV (*n* = 3)	99.7 ± 30.2	48.7 ± 6.8	75.6 ± 25.6	50.5 ± 10.0
*p* Value	0.37	0.47	0.77	0.29
Controls				
Gender				
Male (*n* = 30)	127.1 ± 38.6	50.8 ± 4.6	90.0 ± 26.1	49.8 ± 7.1
Female (*n* = 10)	127.5 ± 33.1	52.9 ± 2.6	81.8 ± 13.7	52.0 ± 1.8
*p* Value	0.97	0.17	0.35	0.12

Note: OI, osteogenesis imperfecta; T4, thoracic vertebra; T10, thoracic vertebra

## Discussion

To our knowledge, this is the first study to compare the differences of muscle size and muscle density between children with OI and controls by opportunistically using low-dose chest CT. We evaluated the trunk muscle differences of muscle size and muscle density between OI patients and control group using the fixed thresholds and the Gaussian mixture model for segmentation of muscle in children. As a main result, the present study demonstrated that muscle density in OI patients was different from controls. Moreover, the fixed thresholds may not be suitable for segmentation of muscle in children.

Bland-Altman limits of agreement that indicate inter-software agreement is within an acceptable range to use either of the two methods. Use of the fixed thresholds to initiate muscle segmentation does not address differences in muscle density caused by variable degrees of muscle fat content, which vary widely among muscles and different ages [17]. Our study indicated that muscle size and muscle density measured by the Gaussian mixture model higher than measured by the fixed thresholds. Previous cross-sectional studies in children have reported significant positive associations between muscle density, which is inversely associated with muscle fat content [14]. Previous study reported that young adults accrued more fat mass at multiple skeletal sites as puberty proceeds [19]. Thus, muscle fat content in children might be different from adults. Moreover, the fixed thresholds used in adults may not be suitable in children.

Previous studies addressed the assessment of extremity muscle size in OI individuals and indicated conflicting results [7, 8]. Veilleux et al. reported that lower limb muscle size was on average 7% smaller in patients with OI than in control participants [7]. Palomo et al. found that forearm muscle size did not differ significantly between OI individuals and controls, but in OI types I and OI type III, forearm muscle size were 8% and 14% lower compared with controls [8]. Lower levels of physical activity and smaller muscle size may have contributed to lower muscle function [7]. Conversely, many patients with severe OI may use their arms for propelling wheelchairs. Thus, the different levels of physical activity may lead to different results in lower limb muscle size and forearm muscle size. However, in a mouse model of OI, evaluation of the muscle fiber size indicated that mice did not have smaller muscle fiber size [20]. Moreover, LoMauroet al reported that cross section area of chest wall was found no difference between OI patients and control group [21]. In this study, we found that there were no differences in the trunk muscle size between OI patients and controls using the fixed thresholds. But there were significant differences in the trunk muscle size using the Gaussian mixture model. Besides, muscle size measured by the Gaussian mixture model higher than measured by the fixed thresholds. These conflicting results may suggest that muscle size may not be a meaningful indication of muscle performance in OI patients.

Our study demonstrated that children with OI had lower T4 muscle density and lower T10 muscle density compared with control group using the Gaussian mixture model and the Gaussian mixture model. However, several studies reported that individuals with OI had normal muscle density compared to age- and sex-matched controls by using peripheral quantitative CT [7, 9, 22]. These studies mainly focused on the muscle differences in the distal lower limb rather than the trunk muscle differences. A possible explanation could be that mutations affecting collagen type I may have a direct effect on muscle, as collagen type I is present in the extra-cellular matrix surrounding muscle fibers, which plays an important role in transmitting muscle force to tendons [23, 24]. Therefore, we may conclude that mutations in collagen type I have a deeper influence in the trunk muscles than in the lower limb. Further studies are needed to confirm this conclusion. The lower muscle density suggests that OI patients have higher muscle fat infiltration that is associated with decreased muscle force [14]. The lipid infiltration of skeletal muscle appears to contribute to age-related decline in skeletal muscle function, which may increase risk of loss of mobility, falls, and skeletal fractures [18]. Previous studies also emphasized the importance of intramuscular fat content and distribution for muscle function [17, 18]. Meanwhile, lower muscle density may indicate that the progressive chest deformities presented by OI patients tend to affect pulmonary function [25]. Therefore, this similarity results may suggest that muscle density measured in the trunk muscles may represent a more clinically meaningful indication of muscle performance and associate with clinical outcomes.

There are several limitations in our study. One important limitation was a retrospective study with a rather small number of patients and limited data. Another limitation of the study was the muscle measurements, which were based on the single slice instead of a full 3D analysis of the complete muscles [12]. Further studies are required to perform the volume segmentation of the muscles.

In conclusion, these results suggest that muscle density may represent a more clinically meaningful indication of muscle performance than muscle size in children with OI. Muscle density measurements of the trunk muscles can be easily obtained from opportunistic low-dose chest CT. Therefore, children with OI had lower trunk muscle density than non-OI children indicating that OI might also impair muscle quality. Moreover, the fixed thresholds may not be suitable for segmentation of muscle in children.

## Data Availability

The datasets used and/or analysed during the current study are available from the corresponding author on reasonable request.

## Abbreviations

OI: Osteogenesis imperfecta
HU: Hounsfield Units
BMI: body mass index
